# Repeated Exposure to Conditioned Fear Stress Increases Anxiety and Delays Sleep Recovery Following Exposure to an Acute Traumatic Stressor

**DOI:** 10.3389/fpsyt.2014.00146

**Published:** 2014-10-20

**Authors:** Benjamin N. Greenwood, Robert S. Thompson, Mark R. Opp, Monika Fleshner

**Affiliations:** ^1^Department of Psychology, University of Colorado Denver, Denver, CO, USA; ^2^Department of Integrative Physiology, University of Colorado Boulder, Boulder, CO, USA; ^3^Center for Neuroscience, University of Colorado Boulder, Boulder, CO, USA; ^4^Department of Anesthesiology and Pain Medicine, University of Washington, Seattle, WA, USA

**Keywords:** REM, NREM, anxiety, diurnal rhythm, conditioning, classical, sleep, chronic stress

## Abstract

Repeated stressor exposure can sensitize physiological responses to novel stressors and facilitate the development of stress-related psychiatric disorders including anxiety. Disruptions in diurnal rhythms of sleep–wake behavior accompany stress-related psychiatric disorders and could contribute to their development. Complex stressors that include fear-eliciting stimuli can be a component of repeated stress experienced by human beings, but whether exposure to repeated fear can prime the development of anxiety and sleep disturbances is unknown. In the current study, adult male F344 rats were exposed to either control conditions or repeated contextual fear conditioning for 22 days followed by exposure to no, mild (10), or severe (100) acute uncontrollable tail shock stress. Exposure to acute stress produced anxiety-like behavior as measured by a reduction in juvenile social exploration and exaggerated shock-elicited freezing in a novel context. Prior exposure to repeated fear enhanced anxiety-like behavior as measured by shock-elicited freezing, but did not alter social exploratory behavior. The potentiation of anxiety produced by prior repeated fear was temporary; exaggerated fear was present 1 day but not 4 days following acute stress. Interestingly, exposure to acute stress reduced rapid eye movement (REM) and non-REM (NREM) sleep during the hours immediately following acute stress. This initial reduction in sleep was followed by robust REM rebound and diurnal rhythm flattening of sleep/wake behavior. Prior repeated fear extended the acute stress-induced REM and NREM sleep loss, impaired REM rebound, and prolonged the flattening of the diurnal rhythm of NREM sleep following acute stressor exposure. These data suggest that impaired recovery of sleep/wake behavior following acute stress could contribute to the mechanisms by which a history of prior repeated stress increases vulnerability to subsequent novel stressors and stress-related disorders.

## Introduction

Although acute activation of the stress response evolved to enhance chances of survival, excessive, chronic, or repeated activation of the stress response can negatively impact central and peripheral physiological systems (1–3) and is a significant risk factor for the development of stress-related mental illness including depression, anxiety, and post-traumatic stress disorder [PTSD; (4–6)]. One way that repeated stressor exposure could facilitate psychiatric disorders is by sensitizing responses to novel stressors. Indeed, individuals with PTSD display exaggerated startle (7–9), autonomic (10, 11), and in some cases hypothalamic–pituitary–adrenal axis (12, 13) responses to aversive stimuli. Similarly, rodents exposed to repeated stressors can display exaggerated hormonal (14–16), autonomic (17, 18) and neuronal (19, 20), responses to acute, novel stressors. These exaggerated responses to novel stressors in rodents can occur concordantly with the development of anxiety- and depression-like behaviors (21, 22).

Disruptions in diurnal rhythms of sleep–wake behavior and specific alterations in sleep architecture accompany stress-related psychiatric disorders and could contribute to their development (23–27). In fact, the Diagnostic and Statistical Manual for Mental Disorders lists sleep disturbance as diagnostic criteria for several types of anxiety disorders, and epidemiological studies reveal that insomnia is a risk factor for depression (28). Sleep architecture can be characterized by measuring stages of sleep using electroencephalogram (EEG). A typical night of sleep consists of episodes of non-rapid eye movement (NREM) sleep, which includes slow-wave sleep and rapid eye movement (REM) sleep. Individuals suffering from panic disorder have disruptions in sleep that include reduced percent (%) time spent in NREM sleep (29, 30). Depressed patients generally display increased % REM, sleep fragmentation, and reduced % NREM (31, 32). Disturbed slow wave (33) and REM (34–37) sleep is also considered a hallmark symptom (38) and post-trauma predictor (39) of PTSD. In rodents, stressor exposure can disrupt REM and NREM sleep and flatten diurnal rhythms of sleep/wake behavior [see Ref. (40, 41) for reviews]. Finally, manipulations of the sleep/wake cycle can alleviate depression symptoms (42, 43). Together, these data suggest that factors that increase vulnerability to sleep disruption could contribute to the development of stress-related psychiatric disorders.

Stressors that are unpredictable and uncontrollable are the most potent in terms of their deleterious consequences on emotion (44) and sleep (41). Rats exposed to a series of uncontrollable tail shocks, but not an equal number and intensity of controllable tail shocks, for example, display behaviors resembling anxiety and depression including a reduction in social exploratory behavior (45), an increase in fear conditioning (46), and a deficit in goal-directed learning in a shuttle-box escape task (44). Exposure to this same uncontrollable stressor can also flatten diurnal rhythms of activity and physiology (47), and increase sleep fragmentation and suppress REM (Thompson et al., unpublished). Similarly, repeated uncontrollable foot shock stress reduces overall time spent in REM in mice during the 20 h period following each uncontrollable shock session or re-exposure to the shock context (48). Interestingly, stress-buffering manipulations such as voluntary exercise prevent both the anxiety- and depression-like behavioral (49–51) and sleep-disrupting consequences of uncontrollable stress in rats. Although it is clear that stress can impact sleep depending on the nature of the stressor (41), whether a history of repeated stressor exposure can sensitize sleep disruption in response to a novel, acute uncontrollable stressor remains unknown.

In the current study, adult male F344 rats were exposed to control conditions or repeated contextual fear conditioning for 22 days followed by exposure to no, mild (10), or severe (100) acute uncontrollable tail shock stress. Complex stressors that include exposure to fear-eliciting stimuli can be a component of repeated stress experienced by human beings. We have previously reported that repeated exposure to fear conditioning can sensitize physiological responses to acute uncontrollable stress and exacerbate uncontrollable stress-induced disruptions in diurnal rhythms of heart rate (HR) and core body temperature (CBT) (18). The goal of the current study was to determine whether repeated exposure to conditioned fear can also prime the development of anxiety and sleep disturbances following exposure to an acute uncontrollable stressor.

## Materials and Methods

### Animals

A total of 141 adult, male F344 rats (Harlan Laboratories) weighing 200–230 g upon arrival were housed under controlled temperature (22°C) and humidity. The animals were maintained on a 12:12 h light/dark cycle (lights on 7:00 a.m. to 7:00 p.m). All rats were single housed in Nalgene Plexiglas cages (45 cm× 25.2 cm× 14.7 cm) and were allowed to acclimate to the housing conditions for 1 week before start of experimental procedures. Rats had *ad libitum* access to food and water and were weighed three times per week. All experimental procedures were performed during the inactive (light) phase of the light:dark cycle and animals were handled during the 1 week acclimation period. Animal discomfort was minimized during all procedures. Experimental protocols for these studies were approved by the University of Colorado Animal Care and Use Committee.

### Repeated fear

Exposure to repeated contextual fear was performed as previously described in detail (18, 52) Briefly, rats were placed into a conditioning chamber (46 cm× 20.7 cm× 20 cm) on day 0 for 5 min in order to acquire a memory of the context, after which rats received three, 2 s, 1.5 mA foot shocks (1 min ITI). This initial contextual fear conditioning occurred at 1000 h. Rats were returned to their home cages immediately following initial conditioning and after every subsequent re-exposure to the conditioned context. Starting 24 h following initial conditioning (day 1), rats were repeatedly exposed to the conditioned context twice a day for 22 days: once in the a.m. and once in the p.m. Each exposure was 20 min in duration and occurred between 8:00 and 12:00 h (a.m. session) and 12:00 and 5:00 h (p.m. session). The time of each a.m. and p.m. exposure was chosen randomly in order to reduce predictability. Freezing, defined by the absence of movement except that required for respiration, was scored during each re-exposure session using a random sampling procedure, whereby rats were either scored as freezing or not freezing every 10 s. To prevent extinction of fear, minimal numbers of re-instatement foot shocks were used to re-instate contextual conditioned fear. When average freezing during an a.m. session fell below 50%, all rats were administered a single re-instatement foot shock (2 s, 1.5 mA) at the end of the p.m. re-exposure session.

### Inescapable tail shock stress

After 22 days of no repeated fear or repeated fear exposure, rats either remained in their home cages and were not exposed to acute stress (0 tail shocks), or were exposed to 10 or 100 inescapable tail shocks as previously described (18). On the day of exposure to tail shock, rats assigned to the tail shock groups were transported to a separate room, placed in Broome-style Plexiglas restraining tubes (23.4 cm long and 7.0 cm in diameter), and exposed to 10 or 100, 5 s, 1.5 mA inescapable tail shocks. Shocks were delivered at a variable-60 s ITI between 8:00 a.m. and 11:00 a.m. Rats were immediately returned to their home cages following termination of the appropriate number of shocks (10 or 100). Inescapable tail shock was used as the acute novel stressor in these experiments because it produces reliable behaviors in rodents resembling human symptoms of stress-related psychiatric disorders (44, 45), and we have previously reported that prior repeated fear stress sensitizes physiological responses to tail shock stress (18).

### Behavioral testing

Measures of juvenile social exploration and shock-elicited freezing were obtained sequentially as previously described (53). Testing for baseline juvenile social exploration occurred 1 week prior to uncontrollable stress. During social exploration testing, each adult experimental subject was placed into separate plastic cages identical to their home cages with bedding and a plastic, filter-top lid between 7:00 a.m. and 8:00 a.m. After 1 h, a 28–32-day-old male juvenile was introduced to the cage for 3 min and exploratory behaviors (sniffing, pinning, and allogrooming) were timed by an observer blind to treatment. After the test, the juvenile was removed and the experimental rat was returned to the home cage. Baseline testing was used to reduce neophobia to the social exploration procedure.

One week after baseline testing, and either 1 or 4 days following 0, 10, or 100 tail shocks, rats were again tested for social exploratory behaviors as described for the baseline test. Different juvenile rats were used for the two social exploration tests, so that experimental rats were not exposed repeatedly to the same juvenile. Social exploration occurred prior to shock-elicited freezing so that shock administered during the fear test would not interfere with social exploration behavior.

Following the completion of social exploration testing, rats were transferred to novel, brightly lit chambers that differed in size, lighting, odor, and background noise from the conditioning chambers used for repeated fear stress. Freezing behavior was observed for 10 min immediately after placement of the rats into the chambers. Rats then received two, 1 s, foot shocks (0.7 mA, 1 min ITI) followed by a 20 min, post-shock freezing observation period. Freezing immediately following shock presentation is a measure of fear conditioned to cues present in the shuttle box (54). Reduction in juvenile social exploration (45, 55) and enhanced shock-elicited freezing (56, 57) represent rodent analogs of social- and fear-related anxiety behaviors, respectively.

### Biotelemetry surgeries

F40-EET biotelemetry transmitters (Data Sciences International, St. Paul, MN, USA) were implanted into animals used in Experiment 3 as previously described (18, 47, 52). Following ketamine (i.p. 75.0 mg/kg), and medetomidine (i.p. 0.5 mg/kg) anesthesia, a midline incision was made approximately 5.0 cm in length on the ventral abdominal wall. Biopotential leads were passed through the ventral abdominal wall and then the transmitter was sutured to the ventral abdominal wall. The EEG leads were placed as previously described in telemetry studies of mice (58, 59). Briefly, insulated leads were passed subcutaneously to the base of the skull, where they were attached to pan head stainless steel screws (Plastics One Inc.), which served as EEG recording electrodes. Screws were placed according to the Rat Brain Atlas in Stereotaxic Coordinates by Paxinos and Watson (60) at anterior 2.0 mm; lateral 2.5 mm and posterior 5.5 mm; lateral 3.0 mm from Bregma using standard stereotaxic methods (61). Screws and leads were embedded in dental acrylic to ensure the integrity of the recording signal. Immediately following surgery, rats were given meloxicam (1.0 mg/kg s.c.) for analgesia after which they recovered on a heating pad at 37°C until ambulatory. Once ambulatory, rats were returned to their home cages and given one 2.0 mg rimadyl tablet (Bio-Serv) and several fruity bites (Bio-Serv). Animals were allowed to recover for 1 week before the start of repeated exposure to conditioned fear.

### Biotelemetry data acquisition and analysis

The F40-EET transmitter (DSI) allows *in vivo* real-time measurement of locomotor activity (LA), HR, CBT, and EEG in freely moving animals. Biotelemetry recordings were acquired/analyzed using Dataquest ART 4.3 Gold Acquisition/Analysis Software (Data Sciences International, St. Paul, MN, USA), as previously described (18, 52). Analyses of the sleep/wake cycles were performed using the automated Neuroscore 2.1.0 software (Data Sciences International, St. Paul, MN, USA). The trace EEG signal was subjected to fast Fourier Transformation (FFT), yielding spectra between 0.5 and 30 Hz in 0.5-Hz frequency bins. The delta frequency band was defined at 0.5–4.5 Hz and the theta frequency band was defined as 6.0–9.0 Hz, as previously described (59). Arousal state was scored in 10-s epochs and classified as NREM, REM, or wake on the basis of state-dependent changes in multiple parameters, including the EEG, LA, HR, and body temperature, as previously described (59, 62). Wakefulness was defined on the basis of a low amplitude, mixed frequency EEG (delta ≈theta) accompanied by body movements (i.e., activity), and increases in body temperature. NREM sleep was identified by increased absolute EEG amplitude with integrated values for the delta frequency band greater than those for the theta frequency and lack of body movements. Body temperature declines upon entry into NREM sleep until it reaches a regulated asymptote. REM was characterized by a low amplitude EEG with integrated values for the delta frequency band less than those for the theta frequency band. Any epochs containing artifact or electrical noise were tagged and excluded from subsequent spectral analysis. All sleep scoring was performed by an individual blind to treatment condition of the animal.

### Experimental design

#### Experiment 1

**Figure 1:**
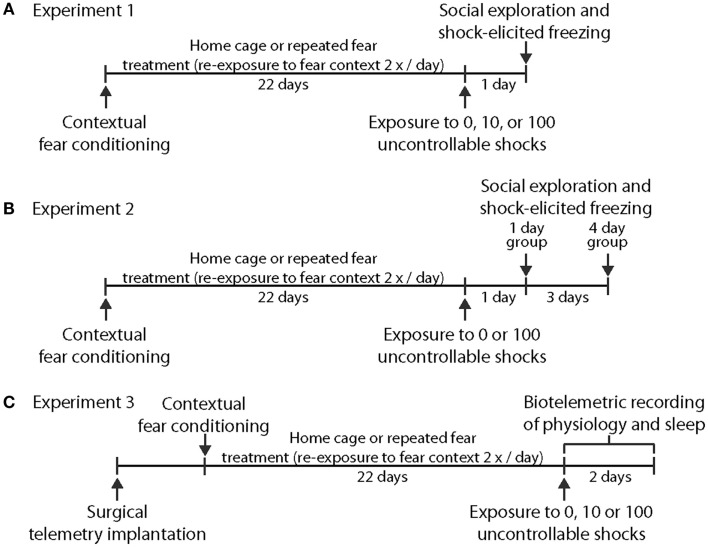
**Time lines depicting the series of events used. in Experiment 1 (A), Experiment 2 (B), and Experiment 3 (C)**.

Experiment 1 was designed to test the hypothesis that prior repeated fear stress sensitizes anxiety responses to novel acute stressor exposure. Rats were randomly assigned to the following groups: no repeated fear (home cage)/0 tail shocks (*n* = 7); home cage/10 tail shocks (*n* = 8); home cage/100 tail shocks (*n* = 8); repeated fear/0 tail shocks (*n* = 8); repeated fear/10 tail shocks (*n* = 8); repeated fear/100 tail shocks (*n* = 7). All rats were tested for anxiety-like behavior using social exploration and shock-elicited freezing 24 h following tail shock exposure. Figure 1A shows the sequence of events followed during Experiment 1.

#### Experiment 2

Experiment 2 explored whether acute stress-induced anxiety-like behavior persisted longer following acute stress in rats previously exposed to repeated fear compared to home cage rats. Rats previously exposed to home cage or repeated fear conditions were exposed to 0 or 100 tail shocks and were then tested for anxiety-like behaviors either 1 or 4 days later. Groups containing home cage and repeated fear rats not exposed to tail shock (the 0 tail shock groups) were split so that half of the rats were tested with the 1-day group and the other half were tested with the 4-day group. No time-dependent effects were noted between the 0 tail shock rats tested at the 1 and 4-day time points, so the time points were combined. Therefore, the home cage/0 tail shock and repeated fear/0 tail shock groups include rats tested at both the day 1 and day 4 time points. The following groups were tested: Home cage/0 (*n* = 8); home cage/1 day after 100 tail shocks (*n* = 8); home cage/4 days after 100 tail shocks (*n* = 8); repeated fear/0 (*n* = 8); repeated fear/1 day after 100 tail shocks (*n* = 7); repeated fear/4 days after 100 tail shocks (*n* = 8). A time line for the procedures used in Experiment 2 can be found in Figure 1B.

#### Experiment 3

Rats implanted with F40-EET transmitters were exposed to home cage or repeated fear conditions for 22 days, followed by 0, 10, or 100 tails shocks. Data were recorded starting 1 week after telemetry implantation and continued throughout the remainder of the experiment. Body weight, freezing behavior during repeated fear stress, HR, body temperature, and activity obtained from the rats used in this experiment have been published previously (18). Here, EEG and physiological data were analyzed starting at clock time 1:00 p.m. (~2 h following the termination of tail shocks) and continued for 3 days thereafter. We waited approximately 2 h following the termination of tail shock stress in order to avoid the disruption in the telemetry signal produced by moving the rats from the stress induction room back to their home cages. Moreover, starting the analyses at the same clock time for all animals eased analyses of the data. Twelve of the initial 48 rats used in the study were dropped from analyses due to loss or interference with the EEG signal, yielding the following groups: home cage/0 tail shocks (*n* = 5); home cage/10 tail shocks (*n* = 7); home cage/100 tail shocks (*n* = 6); repeated fear/0 tail shocks (*n* = 5); repeated fear/10 tail shocks (*n* = 7); repeated fear/100 tail shocks (*n* = 6). A time line for the procedures used in Experiment 3 can be found in Figure 1C.

### Statistical analysis

Body weight data were analyzed using repeated measures ANOVA. Average time spent exploring during the 3 min social exploration test and average % freezing during the 20 min post-shock freezing period in Experiment 1 were analyzed using 2 (home cage, repeated fear) × 3 (0, 10, 100 tail shocks) ANOVAs or, for Experiment 2, 2 (home cage, repeated fear) × 3 (no acute stress, 1 day after 100 tail shocks, 4 days after 100 tail shocks) ANOVAs. Percent time spent in REM, NREM, and wake during the remaining 6 h of the light cycle starting approximately 2 h following termination of acute stress were collapsed into 1 h blocks and analyzed with 2 (home cage, repeated fear) × 3 (0, 10, 100 tail shocks) repeated measures ANOVA. Subsequent percent time spent in REM, NREM, and wake were collapsed into 12 h blocks and compared with 2 × 3 ANOVAs. Light and dark cycles were analyzed independently. Diurnal differences of average % REM, % NREM, and % wake, calculated by subtracting the average dark cycle value from the average light cycle value, were compared using 2 × 3 ANOVAs. Fisher’s PLSD *post hoc* analyses were used as appropriate. Group means were considered different when *p* < 0.05.

## Results

### Freezing data and body weight

**Figure 2:**
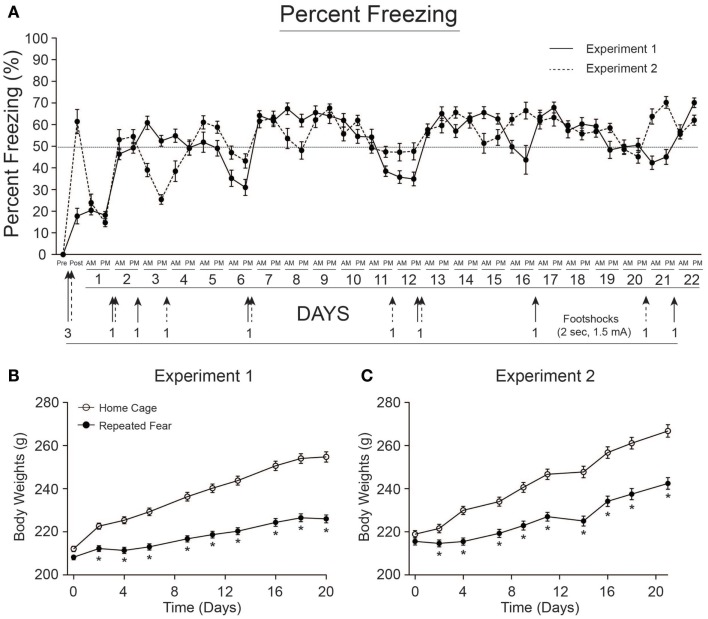
**Freezing behavior and body weights for rats used in Experiments 1 and 2**. **(A)** Freezing was scored before (pre) and after (post) administration of 3 foot shocks during contextual fear conditioning on day 0. Rats were re-exposed to the conditioned context twice a day, early (a.m.), or late (p.m.) during the light cycle, for 22 days. Re-instatement shocks (denoted by arrows) were administered at the end of the p.m. session when average freezing fell below 50%. The number of shocks administered is noted next to each arrow. Rats in both Experiment 1 **(B)** and Experiment 2 **(C)** exposed to repeated fear stress gained less weight over time relative to home cage control rats. *p < 0.05 relative to home cage control.

Rats exposed to repeated conditioned fear stress for 22 days displayed freezing behavior upon each re-exposure to the conditioned context (Figure 2A). Rats in both Experiments 1 and 2 required 6 foot shocks to maintain levels of freezing above 50%. Body weights of rats used in Experiments 1 and 2 are shown in Figures 2B,C, respectively. Both groups gained weight over time [Experiment 1, *F*(8, 352) = 467.1; *p* < 0.0001; Experiment 2, *F*(8, 360) = 704.5; *p* < 0.0001], but rats exposed to repeated fear stress gained less weight over time compared to rats exposed to home cage treatment [Experiment 1, *F*(8, 352) = 57.56; *p* < 0.0001; Experiment 2, *F*(8, 360) = 37.97; *p* < 0.0001]. Freezing and body weight data from rats used in Experiment 3 have been published previously (18) and thus are not shown.

### Prior exposure to repeated fear stress increases anxiety as measured by shock-elicited freezing

**Figure 3:**
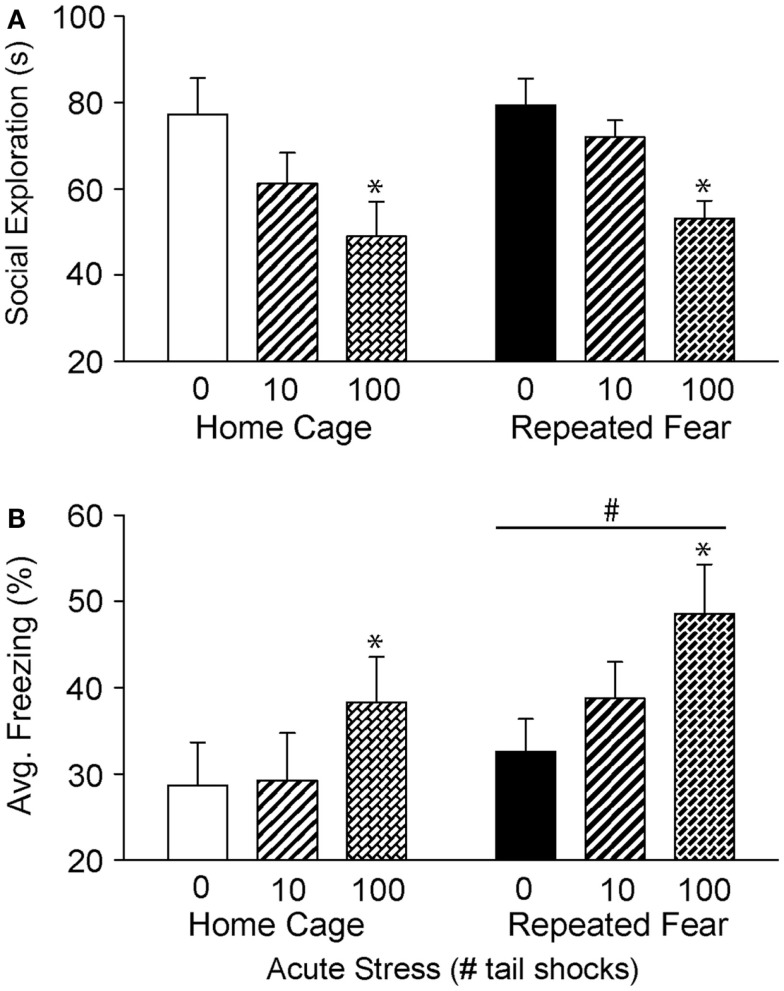
**Effects of prior repeated fear stress on anxiety behavior following mild or severe acute stress**. Following 22 days of no repeated fear (home cage) or repeated fear stress (repeated fear), rats were exposed to no acute stress (0), mild (10 uncontrollable tail shocks; 10), or severe (100 uncontrollable tail shocks; 100) acute stress. Juvenile social exploration **(A)** and shock-elicited freezing **(B)** were measured 24 h later in a novel environment. *p < 0.05 relative to 0 groups; #main effect of repeated fear stress (*p* < 0.05).

Twenty four hours following exposure to 0, 10, or 100 tail shocks, rats used in Experiment 1 were tested for social exploratory behavior and shock-elicited fear. Consistent with prior reports (50, 53, 55), exposure to acute stress reduced social exploration [*F*(2, 40) = 8.48; *p* = 0.0008; Figure 3A] and increased shock-elicited freezing [*F*(2, 40) = 3.54; *p* = 0.03; Figure 3B]. Only 100 tail shocks reduced social exploration (*p* = 0.02) and increased fear (*p* = 0.01). The reduction in social exploration (*p* = 0.07) and the increase in fear (*p* = 0.07) following 10 shocks failed to reach significance. A history of repeated fear had no impact on social exploratory behavior [*F*(1, 40) = 1.13; *p* > 0.05], thus acute stress reduced social exploratory behavior equally regardless of history of repeated fear. In contrast, rats exposed to repeated fear displayed more fear than rats exposed to home cage treatment [*F*(1, 40) = 3.82; *p* < 0.05]. These effects occurred in the absence of gross changes in LA. Neither acute tail shock stress [*F*(2, 40) = 1.97; *p* > 0.05] nor repeated fear [*F*(1, 40) = 1.62; *p* > 0.05] altered the number of spontaneous cage crosses during social exploration testing (data not shown).

**Figure 4:**
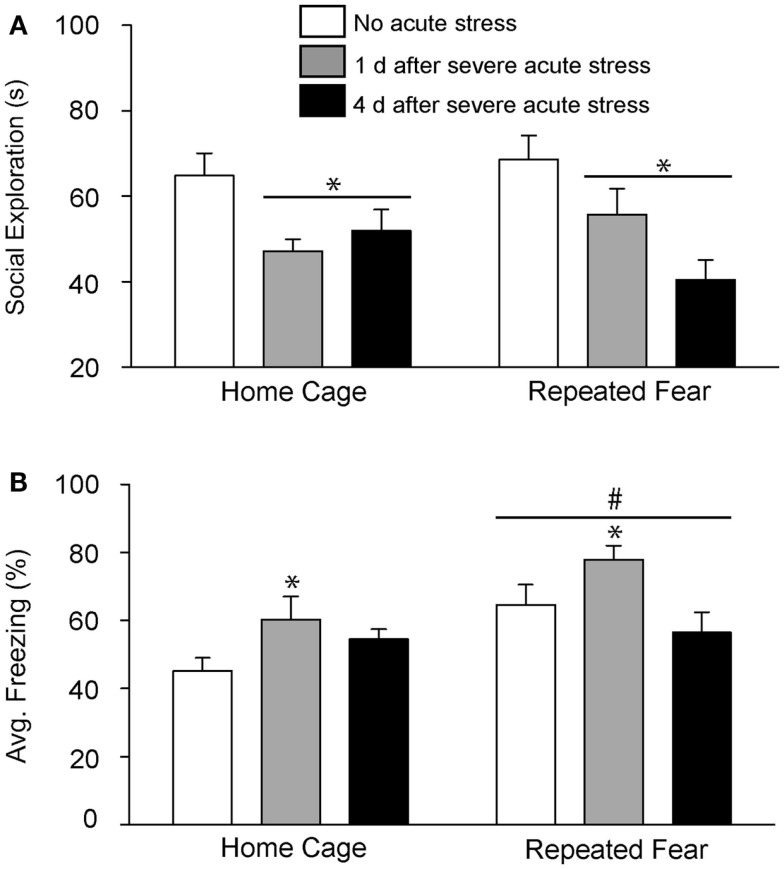
**Effects of prior repeated fear stress on persistence of anxiety behavior following severe acute stress**. Following 22 days of no repeated fear (home cage) or repeated fear stress (repeated fear), rats were either exposed to no acute stress or to 100 uncontrollable tail shocks (acute severe stress). Juvenile social exploration **(A)** and shock-elicited freezing **(B)** were measured either 1 or 4 days later in a novel environment. *p < 0.05 relative to 0 groups; #main effect of repeated fear stress (*p* < 0.05).

In Experiment 2, rats exposed to repeated fear or home cage treatments were tested for anxiety-like behavior either 1 or 4 days following 0 or 100 tail shocks. Results similar to those observed in Experiment 1 were seen here. Exposure to acute stress reduced social exploration [*F*(2, 41) = 9.38; *p* = 0.0004; Figure 4A] and increased shock-elicited freezing [*F*(2, 41) = 4.75; *p* = 0.01; Figure 4B]. Regardless of history of prior repeated fear exposure, a reduction in social exploratory behavior was observed both 1 (*p* = 0.003) and 4 (*p* = 0.0002) days following tail shock stress, whereas the increase in shock-elicited freezing was only present when rats were tested 1 day following tail shock (*p* = 0.01). Although a history of repeated fear had no impact on social exploration [*F*(1, 41) = 0.007; *p* > 0.05], exaggerated shock-elicited freezing was again observed in rats exposed to repeated fear stress [*F*(1, 41) = 9.52; *p* = 0.004]. This exaggerated fear produced by repeated fear stress relative to home cage treatment was temporary. Exaggerated fear produced by repeated fear stress was present in rats not exposed to acute tail shock stress (*p* = 0.01) and 1 day (*p* = 0.02), but not 4 days (*p* > 0.05), following acute tail shock stress. Again, neither acute tail shock stress [*F*(2, 41) = 3.07; *p* > 0.05] nor repeated fear [*F*(1, 41) = 0.16; *p* > 0.05] altered the number of cage crosses during social exploration testing (data not shown).

### Repeated fear prolongs REM and NREM sleep loss immediately following acute stress

**Figure 5:**
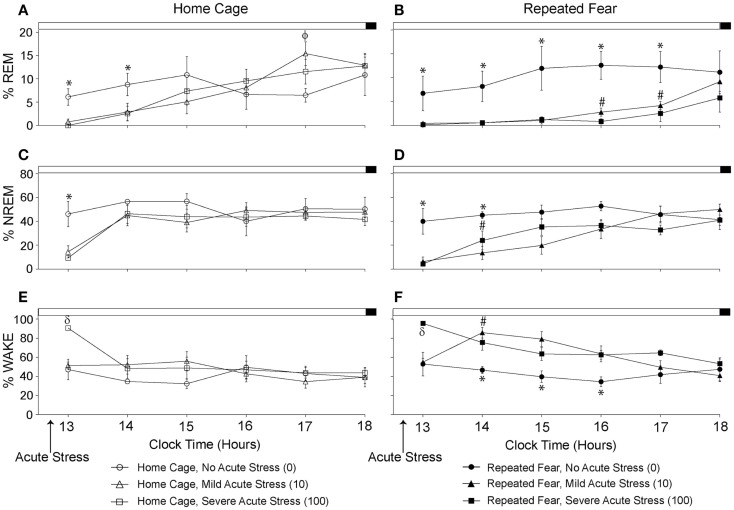
**Effects of repeated fear stress on sleep/wake behavior during the remainder of the light cycle following acute stressor exposure**. Following 22 days of no repeated fear (home cage) or repeated fear stress (repeated fear), rats were exposed to no acute stress (0), mild (10 uncontrollable tail shocks; 10), or severe (100 uncontrollable tails shocks; 100) acute stress. Percent time spent in rapid eye movement [REM; **(A,B)**] sleep, non-rapid eye movement [NREM; **(C,D)**] sleep, and wake **(E,F)** were determined in the home cage during the remainder of the light cycle starting approximately 2 h following the termination of acute tail shock stress. *p < 0.05 relative to respective 10 and 100 groups; #*p* < 0.05 relative to home cage/10 and home cage/100 groups; ϕ*p* < 0.05 relative to home cage/0 group; δ*p* < 0.05 relative to respective 0 and 10 groups.

The % time spent in REM, NREM, and wake during the remaining 6 h of the light cycle starting approximately 2 h following the termination of acute tail shock stress are shown in Figure 5. Acute stress reduced % REM (Figures 5A,B) and % NREM (Figures 5C,D) in both home cage and repeated fear groups during the first few hours following acute stress. The reduction in % REM and % NREM following acute stress persisted longer in rats that had been previously exposed to repeated fear stress compared to home cage treatment. This was especially true for % REM. In contrast to the effect of acute stress in home cage rats, which only persisted for 2 h, both 10 and 100 tail shocks suppressed % REM in repeatedly stressed rats for 5 h. These results were confirmed with repeated measures ANOVA, which revealed a significant main effect of acute stress [*F*(2, 30) = 4.22; *p* = 0.02] and significant interactions between acute stress and time (10, 150) = 2.5; *p* = 0.008) and acute stress, time, and repeated fear [*F*(10, 150) = 2.41; *p* = 0.01] on % REM; and significant main effects of repeated fear [*F*(1, 30) = 5.45; *p* = 0.02], acute stress [*F*(2, 30) = 5.6; *p* = 0.02], and significant interactions between repeated fear and time [*F*(5, 150) = 2.68; *p* = 0.02] and acute stress and time [*F*(10, 150) = 4.29; *p* < 0.0001] on % NREM. The acute stress-induced reduction in REM and NREM sleep was accompanied by a detectible increase in wakefulness (Figures 5E,F). ANOVA revealed significant main effects of repeated fear stress [*F*(1, 30) = 7.32; *p* = 0.01] and acute stress [*F*(2, 30) = 6.9; *p* = 0.005]; and a significant interaction between acute stress and time [*F*(10, 150) = 3.08; *p* < 0.0001]. The home cage and repeated fear groups not exposed to acute stress (the 0 groups) did not differ in any parameter measured. See graphs for results of *post hoc* comparisons.

### Prior exposure to repeated fear impairs REM rebound following acute stress

**Figure 6:**
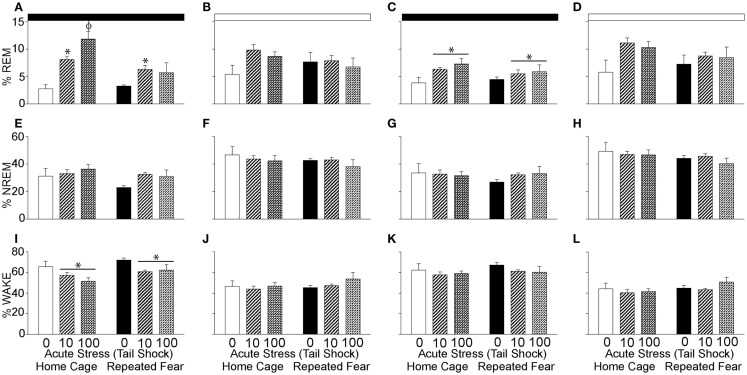
**Effects of repeated fear and acute stress on diurnal pattern of undisturbed home cage sleep/wake behavior**. Following 22 days of no repeated fear (home cage) or repeated fear stress (repeated fear), rats were exposed to no acute stress (0), mild (10 uncontrollable tail shocks; 10), or severe (100 uncontrollable tails shocks; 100) acute stress. Percent time spent in rapid eye movement [REM; **(A–D)**] sleep, non-rapid eye movement [NREM; **(E–H)**] sleep, and wake **(I–L)** were determined in the home cage for 48 consecutive hours starting the dark cycle immediately following the day of acute stressor exposure. Averaged dark and light cycle data are reported in 12 h blocks. *p < 0.05 relative to 0 groups (lines represent main effects of acute stress); ϕ*p* < 0.05 relative to all other groups. Dark bar represents 12 h dark cycle; white bar represents 12 h light cycle.

The % REM, % NREM, and % wake during the 48 h following the beginning of the first night cycle after acute stress are shown in 12 h blocks in Figure 6. Consistent with REM rebound following periods of REM sleep loss (63) and stress (64, 65), acute stress increased % REM during the first [*F*(2, 30) = 14.8; *p* < 0.0001; Figure 6A] and second [*F*(2, 30) = 3.97; *p* = 0.03; Figure 6C] 12 h dark cycles following acute stress. The % REM in the light cycle was not impacted by acute or repeated fear stress (Figures 6B,D); however, the effect of acute stress on % REM in the second light cycle following acute stress just missed significance (*p* = 0.06). Repeated fear impaired the REM rebound that occurred in acute stress groups during the first dark cycle that followed acute stress (Figure 6A). This was confirmed with ANOVA, which revealed a significant main effect of repeated fear [*F*(1, 30) = 8.18; *p* = 0.007] and a significant interaction between acute stress and repeated fear [*F*(2, 30) = 4.75; *p* = 0.01; see Figure 6A for results of *post hoc* tests]. At no time did the home cage and repeated fear groups not exposed to acute stress differ in their % REM. Neither repeated fear nor acute stress altered % NREM (Figures 6E–H).

The increase in % REM following acute stress was paralleled by a reduction in wakefulness (Figures 6I–L). Both acute stress [*F*(2, 30) = 5.59; *p* = 0.008] and repeated fear [*F*(1, 31) = 5.6; *p* = 0.02] increased % wake during the first dark cycle following acute stressor exposure (Figure 6I). At no other time following acute stress was % wake altered by acute or repeated fear stress. The % REM, % NREM, and % wake in all groups resembled control values after the second light cycle that followed acute stress, thus these data are not shown.

### Prior exposure to repeated fear prolongs the flattening of the diurnal rhythm of NREM sleep following acute stress

**Figure 7:**
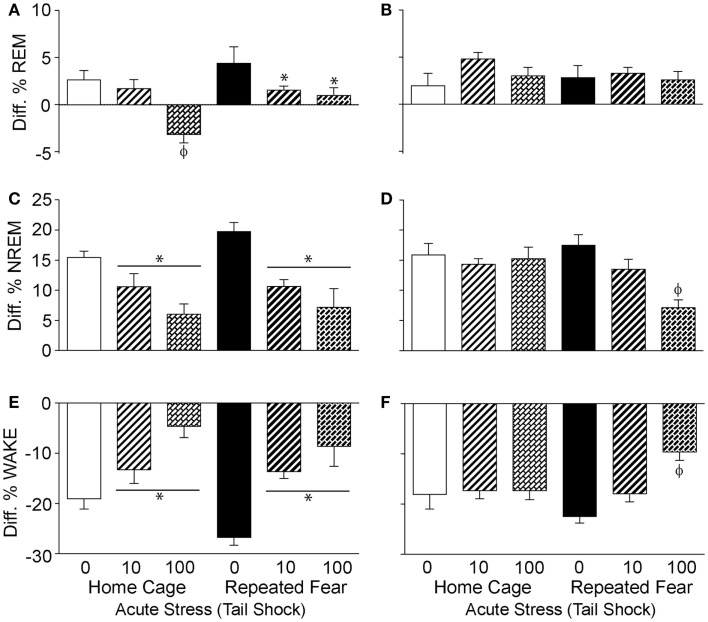
**Effects of repeated fear and acute stress on diurnal difference of sleep/wake behavior**. Following 22 days of no repeated fear (home cage) or repeated fear stress (repeated fear), rats were exposed to no acute stress (0), mild (10 uncontrollable tail shocks; 10), or severe (100 uncontrollable tails shocks; 100) acute stress. Diurnal difference of % rapid eye movement [REM; **(A,B)**] sleep, non-rapid eye movement [NREM; **(C,D)**] sleep, and wake **(E,F)** were calculated by subtracting light cycle values from dark cycle values of sleep parameters measured in the home cage for 48 h starting the dark (active) cycle immediately following acute stressor exposure. The diurnal differences calculated from the first 24 h period starting the first active (dark) cycle immediately following acute stress are shown in the left panel **(A,C,E)**. The diurnal differences calculated from the second 24 h period following acute stress are shown in the right panel **(B,D,F)**. *p < 0.05 relative to respective 0 groups (lines represent main effects of acute stress); ϕ*p* < 0.05 relative to all other groups.

To determine the impact of repeated fear and acute uncontrollable stress on the diurnal rhythms of sleep/wake behavior, the diurnal difference (dark–light) of % REM, % NREM, and % wake were compared between groups following acute stressor exposure. Consistent with greater REM rebound in the light cycle observed in the home cage rats following acute stress (Figure 6A), both repeated fear [*F*(1, 30) = 5.67; *p* = 0.02] and acute stress [*F*(2, 30) = 10.37; *p* = 0.0004] reduced the diurnal difference of % REM (Figure 7A). The flattening of the diurnal rhythm of % REM sleep was present during the first, but was gone by the second (Figure 7B), 24 h period following acute stress. Acute stress reduced the diurnal difference of % NREM sleep [*F*(2, 30) = 14.3; *p* < 0.0001; Figure 7C] and % wake [*F*(2, 30) = 18.77; *p* < 0.0001; Figure 7E] during the first 24 h period following acute stress in both home cage and repeated fear-exposed rats. During the second 24 h period following acute stress, significant interactions between repeated fear and acute stress revealed that the flattening of the diurnal rhythm of both % NREM sleep [*F*(2, 30) = 4.88; *p* = 0.01; Figure 7D] and % wake [*F*(2,30) = 5.44; *p* = 0.009; Figure 7F] persisted longer following acute stress in rats previously exposed to repeated fear. See Figure 7 for results of *post hoc* tests.

## Discussion

Here, we report that prior exposure to a repeated emotional stressor can increase anxiety-like behavior and induce prolonged sleep disruption following exposure to a subsequent acute, novel stressor. These observations add to our prior observations that repeated exposure to conditioned fear stress can sensitize HR and CBT responses to acute severe stress (18). Taken together, these data indicate that repeated exposure to conditioned fear stress can produce behaviors in rodents that resemble characteristics of stress-related psychiatric disorders, including sensitized autonomic responses (18), enhanced fear learning (Figures 3B and 4B), and prolonged stress-induced flattening of biological rhythms including NREM sleep (Figure 7D) following exposure to a novel, acute uncontrollable stressor.

Exposure to severe (100 tail shocks), but not mild (10 tail shocks), acute uncontrollable stress reduced social exploratory behavior and increased freezing immediately following administration of foot shocks during fear conditioning in a novel environment (Figures 3 and 4). The observation that mild acute stress was insufficient to elicit anxiety-like behavior is consistent with prior work showing that greater than 50 uncontrollable tail shocks are required to activate serotonin (5-HT) neurons in the dorsal raphe nucleus (66); the putative mechanism by which acute severe stress transiently enhances fear conditioning (67, 68) and reduces social exploration (45). Repeated fear stress neither sensitized (Figure 3A) nor prolonged (Figure 4A) the effect of acute uncontrollable stress on social exploration. In contrast, a history of repeated fear stress enhanced shock-elicited freezing, regardless of acute stressor exposure (Figures 3B and 4B). These data indicate that like a history of acute stress (50, 69, 70), prior exposure to repeated fear stress can enhance the acquisition of a new fear memory. Similarly, clinical data indicate that patients with PTSD respond in an exaggerated manner to novel fear-eliciting stimuli and remember these aversive stimuli better than trauma-exposed controls without PTSD (10).

The fact that repeated fear stress increased anxiety-like behavior as measured by shock-elicited freezing but not social avoidance suggests that repeated fear stress impacts various anxiety-related behaviors differently. It is unlikely that a history of foot shock during repeated fear sensitized the anxiety response to subsequent foot shock during anxiety testing, because repeated exposure to homotypic stimuli typically lead to habituation, not sensitization, of the stress response to that stimuli (20, 71). Although the exaggerated fear and social avoidance produced by acute uncontrollable stress are thought to have similar mechanisms involving 5-HT and the amygdala (55, 72), repeated fear stress might impact a neural substrate capable of modulating fear- and not social-related anxiety behaviors. Although the hippocampus is an attractive candidate because of its involvement in contextual fear conditioning (73) and sensitivity to repeated stress (74), enhanced fear learning produced by repeated fear stress was observed immediately following shock administration during conditioning in a novel context, a time during which freezing is independent of the hippocampus (75). The prefrontal cortex is an important emotional control region that is known to undergo structural remodeling following repeated stress (76, 77) and can modulate fear expression (78). Thus, the prefrontal cortex could be a structure through which repeated fear acts to enhance fear-related anxiety-like behavior. Consistent with this possibility are the observations of functional and structural deficits in the PFC of patients suffering from PTSD and depression (79–82).

Rats exposed to repeated conditioned fear had similar sleep patterns to rats exposed to home cage treatment in the absence of tail shock (the acute stress 0 groups), suggesting that repeated exposure to conditioned fear may not by itself impact sleep. This is surprising considering that both re-exposure to a cue (83) or a context (84) previously associated with a foot shock has been reported to reduce % REM sleep during the inactive cycle. One reason for this discrepancy could be that the impact of fear conditioning on sleep habituates with time. Indeed, Kant et al. (85) reported that although total sleep time is initially reduced during repeated daily exposure to foot shock stress in the home cage, total sleep time returns to baseline levels by the 7th day of stress. This explanation seems unlikely, however, considering that (1) the return to baseline sleep time following repeated foot shock reported in Ref. (85) was accompanied by a disruption of the normal diurnal rhythm of sleep and no such diurnal disruption following repeated fear stress was observed in the current study and (2) physiological and fear responses to the conditioned context were maintained throughout the duration of the study (18). Moreover, although extinction of fear reduces the impact of re-exposure to a contextual conditioned stimulus on REM sleep (84), fear extinction was prevented in the current study by administration of foot shocks when average freezing levels dropped below 50%. An alternative explanation could be the strain of rat used in the study. Tang et al. (64) reported that relative to Lewis and Wistar rats, which exhibit a reduction in % REM following re-exposure to a conditioned context, F344 rats display no such reduction. In fact, F344 rats displayed the greatest fear response and an increase in % REM during the dark cycle following both acquisition and expression of contextual fear conditioning (86). The possibility remains, however, that repeated fear did disrupt sleep, but that disrupted sleep had resolved by the time analyses of sleep patterns began 24 h following the last exposure to the contextual CS. Indeed, Moreau et al. (87) report that changes in % REM sleep produced by several weeks of mild stress disappeared progressively following termination of stress (87).

In contrast to the lack of observed effect of repeated fear stress on sleep, exposure to acute tail shock stress clearly impacted sleep. Similar to prior reports (88), acute stress reduced both % REM and % NREM sleep and increased % wake during the hours immediately following acute stressor exposure. These data are consistent with our prior work reporting that this same stressor increases activity, HR, and CBT for several hours following stress (18, 47). Compared to home cage rats exposed to acute stress, rats previously exposed to repeated fear displayed a prolonged reduction in % REM and % NREM sleep for hours following stress. In fact, rats not exposed to repeated fear began to show signs of REM recovery by 5 h post-acute stress (Figure 5), a time point during which % REM of rats exposed to repeated fear stress was still suppressed. Similar to the effect of acute stress observed in the current study, victims of acute traumatic injury display increased wake time following trauma (39). Interestingly, those trauma victims who also have disrupted REM sleep (increased frequency but very short duration REM sleep bouts) were also more likely to develop PTSD (39). Consistent with these clinical data, acute stress impacted REM sleep of rats previously exposed to repeated fear to a greater extent than home cage control rats, and this suppressed REM sleep was associated with the most robust shock-elicited freezing.

Rapid eye movement rebound [increased REM sleep after periods of REM suppression; (63)] has been reported to follow stressor exposure (64) and has been argued to represent an adaptive response to stress [for a review, see Ref. (65)]. Significant REM rebound was observed in home cage rats during several dark periods following acute stressor exposure (Figures 5A and 6C). In contrast, and despite the prolonged loss of REM sleep produced by acute stress in the rats exposed to repeated fear, rats exposed to repeated fear had impaired REM rebound (Figure 6A). Suppressed REM rebound following acute stress in rats exposed to repeated fear could thus represent a maladaptive response to acute novel stress that could contribute to vulnerability to stress-related disorders.

The mechanisms underlying REM rebound following stress have been reviewed (65) and could involve serotonin (88), corticosterone (89), prolactin (65, 90), or the central nucleus of the amygdala (91). Further work will be required to elucidate the mechanisms by which repeated fear exposure impairs REM rebound. It should be mentioned, however, that repeated fear stress does not result in exaggerated corticosterone responses to acute tail shock stress (18). Thus, differences in circulating corticosterone are unlikely to be involved in the observed suppression of the REM rebound.

In addition to prolonging sleep disruption immediately following acute stress and suppressing REM rebound, repeated fear stress produced a protracted disruption in the diurnal rhythm of NREM sleep elicited by acute stress. We have previously reported that prior repeated fear stress prolongs the acute stress-induced disruption of the diurnal rhythms of HR and CBT (18). Here, we extend those observations to include disruptions in diurnal rhythms of NREM. Although no differences in total % NREM were observed following exposure to repeated or acute stress (Figure 6), prolonged damping of diurnal rhythms could itself be a precursor to mood disorders (92, 93). Indeed, disruption of sleep/wake cycles, such as occurs with seasonal affective disorders (94) and shift work (95, 96), can trigger mood-related problems in vulnerable individuals.

In conclusion, we present evidence that exposure to repeated fear stress increases selective measures of anxiety-like behavior, prolongs sleep disruption including REM and NREM suppression and NREM diurnal disruption, and impairs REM rebound following exposure to an acute, novel stressor. Sensitization of sleep disruption following acute stressors could contribute to the mechanisms by which a history of repeated stress leads to vulnerability to stress-related psychiatric disorders including anxiety.

## Author Contributions

Benjamin N. Greenwood and Robert S. Thompson conducted experiments, collected data, and co-authored the manuscript. Benjamin N. Greenwood, Robert S. Thompson, Mark R. Opp, and Monika Fleshner designed the experiments and analyzed data. Mark R. Opp and Monika Fleshner edited the final manuscript.

## Conflict of Interest Statement

The authors declare that the research was conducted in the absence of any commercial or financial relationships that could be construed as a potential conflict of interest.
